# Synthesis and Pharmacological Evaluation of Novel 1-(1,4-Alkylaryldisubstituted-4,5-dihydro-1*H*-imidazo)-3-substituted Urea Derivatives

**DOI:** 10.3390/molecules21050582

**Published:** 2016-04-30

**Authors:** Elżbieta Szacoń, Marzena Rządkowska, Agnieszka A. Kaczor, Ewa Kędzierska, Jolanta Orzelska-Górka, Sylwia Fidecka, Dariusz Matosiuk

**Affiliations:** 1 Department of Synthesis and Chemical Technology of Pharmaceutical Substances with Computer Modeling Lab, Faculty of Pharmacy with Division of Medical Analytics, Medical University of Lublin, 4a Chodźki St., Lublin PL-20093, Poland; marzena.rzadkowska@umlub.pl (M.R.); agnieszka.kaczor@umlub.pl (A.A.K.); 2 School of Pharmacy, University of Eastern Finland, Yliopistonranta 1, P.O. Box 1627, Kuopio FI-70211, Finland; 3 Department of Pharmacology and Pharmacodynamics, Faculty of Pharmacy with Division of Medical Analytics, Medical University of Lublin, 4A Chodźki St., Lublin PL-20093, Poland; ewa.kedzierska@umlub.pl (E.K.); jolanta.orzelska@umlub.pl (J.O.-G.); sylwia.fidecka@umlub.pl (S.F.)

**Keywords:** 1-alkyl-4-aryl-4,5-dihydro-1*H*-imidazo-2-amines, antinociceptive compounds, central nervous system (CNS) activity, *in silico* ADMET studies

## Abstract

Novel 1-(1,4-alkylaryldisubstituted-4,5-dihydro-1*H*-imidazo)-3-substituted urea derivatives have been synthesized and evaluated for their central nervous system activity. Compounds **3a**–**m** were prepared in the reaction between the respective 1-alkyl-4-aryl-4,5-dihydro-1*H*-imidazol-2-amines **1a**–**c** and appropriate isocyanates **2** in dichloromethane. The compounds were subjected to *in silico* ADMET studies in order to select best candidates for *in vivo* experiments. The effects of the compounds on the spontaneous locomotor activity and amphetamine-evoked hyperactivity were estimated. Analgesic activity, without or in the presence of naloxone, was assessed in the writhing test. The tendency to change the HTR, evoked by l-5-HTP and the involvement in alteration in body temperature in mice was studied. Additionally, to check possible occurrence of drug-induced changes in the muscle relaxant activity of mice, which may have contributed to their behaviour in other tests, the rota-rod and chimney tests were performed. The new urea derivatives exerted significant activities in the performed pharmacological tests, although the presented results show a preliminary estimation, and thus, need to be extended for identification and understanding the complete pharmacological profile of the examined compounds.

## 1. Introduction

**Figure 1:**
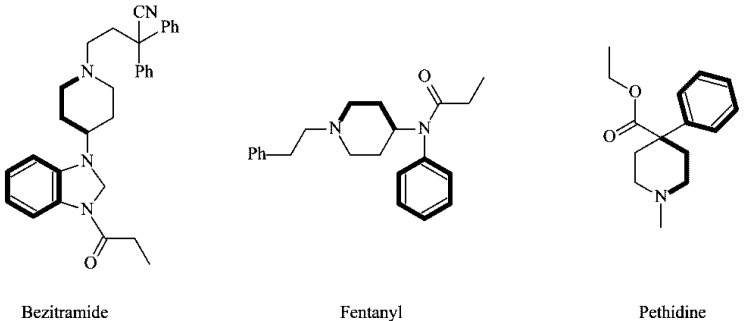
Structural formulas of bezitramide, fentanyl and pethidine. Pharmacophoric features according to Beckett’s model are shown in bold.

Pain is a global health challenge with social and economic implications. At present, the main pain management strategies are administration of opioids and nonsteroidal anti-inflammatory drugs (NSAIDs). Thus, opioid receptors are among the most important molecular targets for antinociceptive medications. Opioid analgesics represent the front-line treatment for the clinical management of moderate to severe pain, and for centuries opium and its extracts have been used for therapeutic purposes [1]. Morphine isolated from opium is one of the most widely used analgesics today. Many morphine-like opioid analgesics have similar structural features, i.e., the phenyl ring, tertiary nitrogen atom and the two carbon fragment (e.g.) as a part of the piperidine ring), required for the receptor fit [2,3,4]. These shared structural features can be found in bezitramide, fentanyl and pethidine, and their analogues, Figure 1 [2]. Non-classical pharmacophore models explaining opioid receptor activity which consist of a base, a hydrophobic and aromatic moiety or hydrogen bond acceptor, hydrophobic, and aromatic groups were also suggested [2,5,6,7,8].

**Figure 2:**
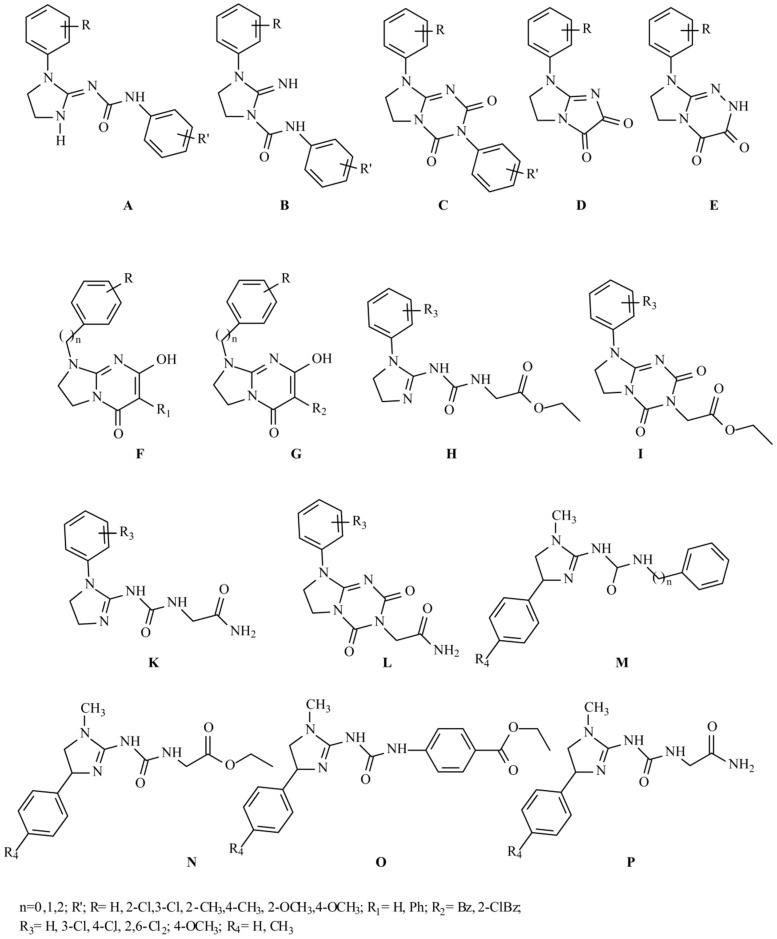
Previously reported antinociceptive and serotoninergic compounds. Series A–E and partially F exert their antinociceptive activity through the opioid system. Series G–P and some of compounds from series F have antinociceptive activity of unknown mechanism [2,6,7,8,9,10,11,12].

Treatment potential and demand for improved opioid analgesics have resulted in development of a number of new opioid analgesics [1]. Based on the non-classical pharmacophore models for opioid receptor activity we have previously reported a few series of compounds with antinociceptive activity mediated through the opioid system (series A–E, Figure 2), partially mediated through opioid system (series F) or with a different mechanism of antinociceptive activity (series G–P) [2,6,7,8,9,10,11,12].

**Scheme 1:**
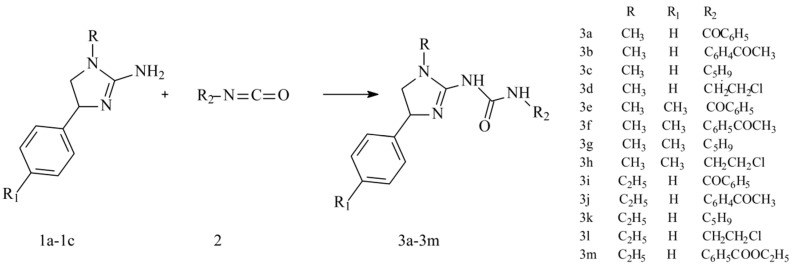
The synthesis scheme of the investigated compounds.

In our continuous effort towards the design and discovery of new antinociceptive compounds we have now designed and synthesized a series of 13 novel 1-(1,4-alkylaryldisubstituted-4,5-dihydro-1*H*-imidazo)-3-substituted urea derivatives **3a**–**m**, (Scheme 1). The rationale of this work can be summarized as follows: (1) the designed compounds follow the non-classical pharmacophore model for opioid receptor activity; (2) the set of substituents in the aryl ring was selected on the basis of our earlier experience with the substituent effect on the activity. Here we present the synthesis, drug-likeness evaluation, ADMET prescreening and pharmacological studies for central nervous system (CNS) activity for 13 novel 1-(1,4-alkylaryldisubstituted-4,5-dihydro-1*H*-imidazo)-3-substituted urea derivatives.

## 2. Results and Discussion

### 2.1. Chemistry

Compounds **3a**–**m** were prepared by the reactions between the respective 1-alkyl-4-aryl-4,5-dihydro-1*H*-imidazol-2-amines **1a**–**c** and the appropriate isocyanates **2** in dichloromethane (Scheme 1). We have previously reported a few series of 1-aryl-4,5-dihydro-1*H*-imidazo-2-amine derivatives which do not possess a protonable nitrogen atom, but exhibit serotoninergic and antinociceptive activity mediated [2,8,10,11,12] or not [6,7] thought the opioid system.

Estimation of drug-likeness and prediction of ADMET properties, performed in order to select best compounds for *in vivo* experiments are presented in the Supplementary Files (Table S1. Parameters for drug-likeness estimation; Table S2. ADMET parameters of the studied compounds; Figure S1. Evaluation of ADMET properties of the studied compounds.)

### 2.2. Pharmacological Activity

Three new urea derivatives named **3a**, **3e**, **3m** were tested for preliminary estimation of their behavioural effects. In the present study a variety of behavioural tests were conducted. At the beginning of the study the toxicity of new compounds was calculated as the ED_50_ based on the loss of the righting reflex within 48 h It was found out that the tested compound **3a** was characterized by moderate toxicity (330.2 mg/kg), whereas compounds **3e** and **3m** had low toxicity (1133.7 mg/kg and 2000 mg/kg, respectively) [13]. These values of ED_50_ were adopted and the regressive doses of ED_50_ were used for further studies (Table 1).

**Figure 3:**
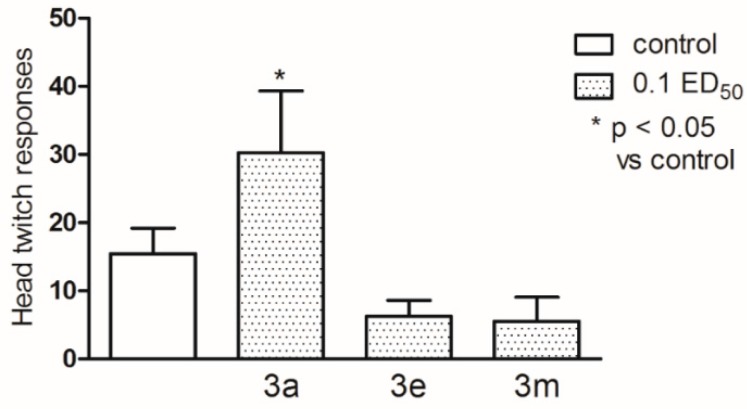
Effect of compounds **3a**, **3e** and **3m** on ‘head-twitch’ responses evoked by l-5-HTP (230 mg/kg) is presented in Figure 1. One-way ANOVA showed significant changes in the number of HTR (F_(3,31)_ = 4.328; *p* < 0.05). *Post hoc* Student’s *t*-test confirmed a significant increase in HTR of mice after administration of the compound **3a** at the dose of 0.1 ED_50_ (*p* < 0.05); * *p* < 0.05 *vs* control.

The HTR elicited by l-5-HTP were evaluated to determine the possible link between the serotonin system and the mechanism of action of the new compounds. These reactions occur as a result of increased activity of central 5-hydroxytryptamine (5-HT) neuronal systems [14] and are caused probably by stimulation of postsynaptic serotonin 5-HT_2_ receptors [15]. Several studies have established that direct and indirect 5-HT agonists induce HTR in rodents [16,17,18,19,20,21,22,23]. Furthermore, 5-HT_2_ receptor antagonists selectively block HTR [24,25,26,27,28,29,30,31,32,33], and their potency is highly correlated with the antagonist’s affinity for 5-HT2 receptors [23,34]. However, this test is not very specific because compounds such as cannabinoids, and benzodiazepines may also cause HTR in animals and other compounds, e.g., adrenergic ligands, can change the HTR [24]. L-5-HTP is the precursor of serotonin, which passes through the blood-brain barrier and in the CNS is decarboxylated to give 5-HT. This leads to an excessive accumulation of 5-HT in the synapstic space, resulting in an increase in its neurotransmission, and production of HTR [28]. All tested compounds showed marked effects on the HTR. Compound **3a** significantly increased the number of HTR, and the compounds **3e** and **3m** caused a reduction of HTR, however, probably due to the large divergence of the data in the studied groups, the results were not statistically significant (Figure 3).

**Figure 4:**
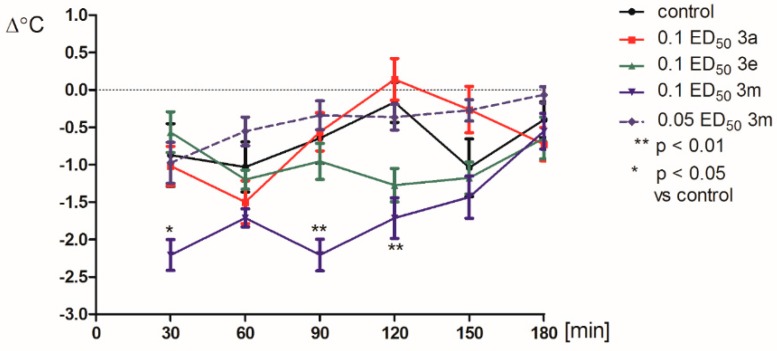
Effect of compounds **3a**, **3e** and **3m** on the body temperature of mice is shown in Figure 2 Two-way ANOVA revealed statistically significant effects of the compounds (F_(4,243_ = 22.55; *p* < 0.0001) and time (F_(5,243)_ = 5.92; *p* < 0.0001) as well as a statistically significant dose x time (F_(20,243)_ = 2.81; *p <* 0.0001). Bonferroni’s *post hoc* test confirmed a significant decrease in the body temperature of mice after the administration of compound **3m** at the dose of 0.1 ED_50_ in 30 (*p <* 0.05), 90 and 120 (*p <* 0.01) min; * *p* < 0.05 *vs* control; ** *p* < 0.01 *vs* control.

The results obtained in this test demonstrate that the mechanism of action of the new compounds may be associated with the serotonin system, however, this correlation is unclear and needs further investigation. On the other hand some confirmation of this hypothesis comes from the body temperature measurement results of the animals. It was shown that the greatest influence on this parameter was related to the compound **3m**, which given at a dose of 0.1 ED_50_ caused a marked reduction in body temperature of the animals up to 120 min of observation. Statistically significant results were obtained at 30 min (*p* < 0.05), and from 90 to 120 min (*p* < 0.01). However, this compound, administered at a twice less dose (0.05 ED_50_) already did not show such activity and the body temperature of mice was similar to the control group. Administration of other compounds **3e** and **3m** practically did not affect the body temperature of animals (Figure 4).

The most important role in the regulation of body temperature play hypothalamic receptors whose action depends, *inter alia*, on serotonin. According to the literature data, the hypothermic effect may be caused by substances which are agonists of serotonin 5-HT_1A_ receptor (such as 8-OH-DPAT) as well as 5-HT_2A_ receptor antagonists (e.g., ketanserin) [34]. Thus, the mechanism of action connected with the serotonin system appears to be most likely for the compound **3m**, which strongly reduced the body temperature of animals and intensely, although not significantly, reduced the number of HTR induced by administration of a serotonin precursor.

Among the compounds tested, only derivative **3e** substantially protected mice against clonic seizures occurring after administration of PTZ. Protection against tonic convulsions and death were also observed, but the effects were not statistically significant. This result is consistent with previous research showing potential anticonvulsant activity of urea derivatives [29]. It may then be assumed that compound **3e** can penetrate across the blood-brain barrier, has lipophilic character and considerably affect CNS. This result may also be another fact proving the possibility of linking the mechanism of action of the new compounds with serotonin system. Animal studies have shown that the administration of substances increasing serotonin levels in the brain (e.g., d-fenfluramine) significantly reduces the incidence of tonic convulsions and completely inhibits mortality, which are due to administration of PTZ. This indicates the important role of serotonin in the protection against seizures induced by this epileptogenic substance.

**Figure 5:**
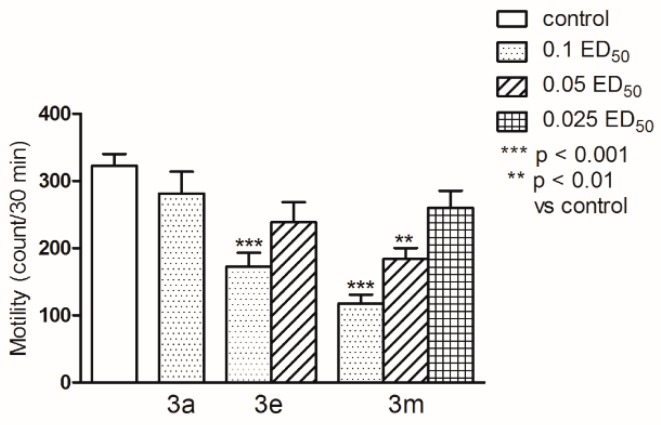
Effect of compounds **3a**, **3e** and **3m** on the spontaneous locomotor activity of mice is given in Figure 3. One-way ANOVA showed significant changes in locomotor activity of mice (F_(6,63)_ = 9.467; *p <* 0.0001). Dunnett’s *post hoc* test confirmed a significant reduction in motility of mice after the administration of tested compound **3e** at the dose of 0.1 ED_50_; (*p* < 0.001) and **3m** at the dose of 0.1 ED_50_ (*p* < 0.001) and 0.05 ED_50_ (*p* < 0.01); ** *p* < 0.01 *vs* control; *** *p* < 0.001 *vs* control.

**Figure 6:**
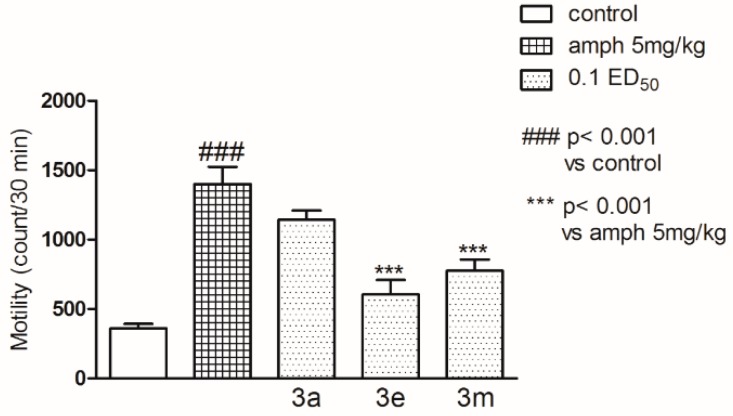
Effect of compounds **3a**, **3e** and **3m** on the amphetamine-induced hyperactivity of mice is presented in Figure 4. One-way ANOVA showed significant changes in locomotor activity of mice (F_(4,43)_ = 20.32; *p <* 0.0001). Dunnett’s *post hoc* test confirmed a significant decrease in motility of mice after the concomitant administration of the compound **3e** and **3m** (*p <* 0.001) with amphetamine 5 mg/kg at the dose of 0.1 ED_50_ vs amphetamine-treated group ### *p* < 0.01 *vs* control; *** *p* < 0.001 *vs* amph 5mg/kg.

Compounds **3e** and **3m** significantly reduced both locomotor activity and amphetamine-induced hyperactivity (Figure 5 and Figure 6). Such impact on the motility of animals may be evidence for the involvement of the catecholamine system in their mechanism of action. A special role is played by adrenergic and dopaminergic receptors. Stimulants, such as D-amphetamine, enhance the release of dopamine in the CNS and induce locomotor activation in mice. The involvement of the brain noradrenergic and dopaminergic system in the behavioural effects of D-amphetamine has been investigated by several groups [31,32,33,34,35,36,37,38,39]. The results obtained in this study may thus indicate the potential use of new compounds in the treatment of some neuropsychiatric disorders associated with excessive activation of the dopaminergic system, although at this stage of investigations, it's just speculation, which must be supported by further research.

The antinociceptive properties of new compounds were tested by performing “writhing” tests. This test is a chemical method useful for sifting molecules whose pharmacodynamics properties are unknown and one of the most sensitive methods to determine the antinociceptive properties; by its use it is possible to detect even very weak antinociceptive agents.

It is also considered as an experimental model closest to the nature of clinical pain. It allows evaluation of analgesic action of both peripheral and central origin; based on chemical stimulation by intraperitoneal administration of agents that irritate serous membranes and provokes characteristic abdominal contractions [40,41]. However, this method has some limitations as in this test, it is difficult to determine the length of antinociceptive activity, and the test is not specific—it can show an analgesic effect for many substances [13,42]. The specificity of the writhing test can be improved by undertaking a preliminary rota-rod test to detect and eliminate molecules that alter the motor performance of animals. In our study it was estimated that none of examined compounds impaired motor coordination in mice.

**Figure 7:**
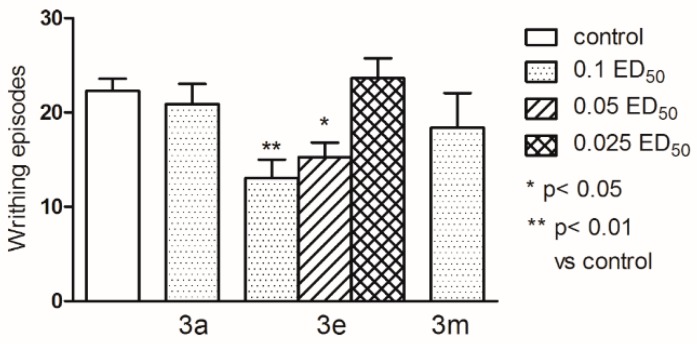
The results of the “writhing” test are given in Figure 5. One-way ANOVA showed significant changes in the number of writhing episodes of mice after the administration of tested compounds (F_(5,68)_ = 4,23; *p <* 0.01). Dunnett’s *post hoc* test confirmed a significant reduction in the writhing episodes of mice after the administration of the compound **3e** at the doses of 0.1 and 0.05 ED_50_ (*p <* 0.01 and *p <* 0.05); * *p* < 0.05 *vs* control; ** *p* < 0.01 *vs* control.

Strong impact on the behaviour of mice in this test was observed for compound **3e**, which administered both in higher dose (0.1 ED_50_) and at twice less dose (0.05 ED_50_) caused very clear and statistically significant (respectively *p* < 0.01 and *p* < 0.05) reduction in the number of writhing episodes in mice (Figure 7).

**Figure 8:**
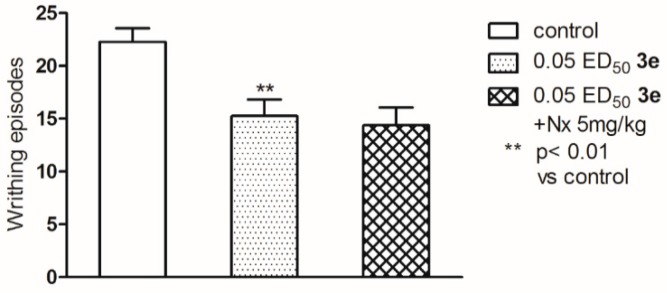
The effect of Nx on the antinociceptive effects of compound **3e** assessed in the “writhing” test in mice. One-way ANOVA showed significant changes in the number of writhing episodes of mice after the administration of compounds **3e** and co-administration of Nx with this compound (F_(2,39)_ = 8.507; *p <* 0.001). *Post hoc* Dunnett’s test showed a significant reduction in the writhing episodes of mice after the administration of the compound **3e** at the dose of 0.05 ED_50_ (*p* < 0.001). Pretreatment with naloxone did not affect the number of writhing episodes as compared to the compound **3e** alone, ** *p* < 0.01 *vs* control.

In order to more closely determine the mechanism of antonociceptive action, the writhing test was performed with the use of nonselective opioid antagonist - naloxone. Naloxone applied at a dose of 5 mg/kg had no effect on the antinociceptive effect of compound **3e** (Figure 8), thus observed activity of compound **3e** is not mediated through the opioid system.

**Figure 9:**
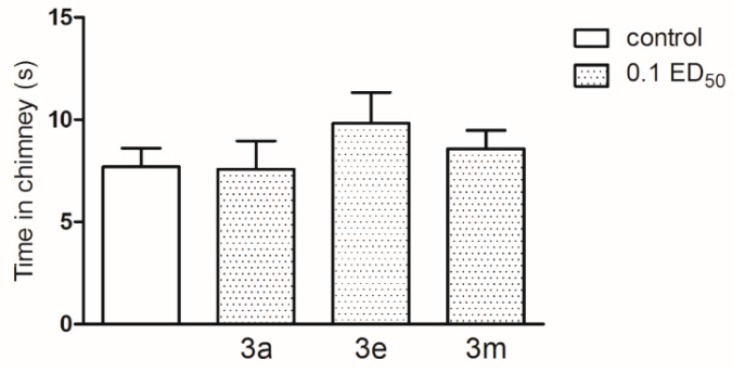
The effect of compounds **3a**, **3e** and **3m** on coordination impairment as measured in chimney test.

**Figure 10:**
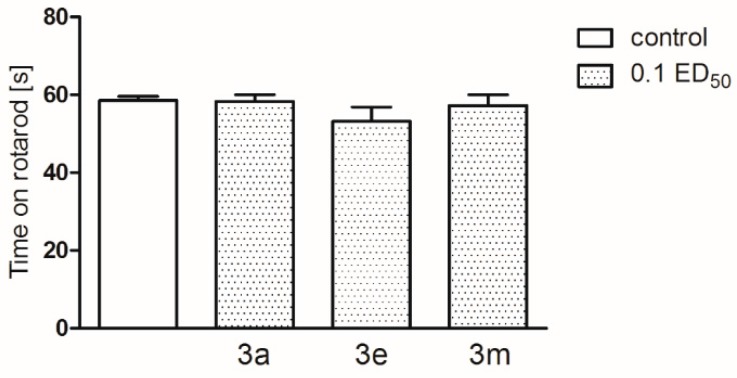
The effect of compounds **3a**, **3e** and **3m** on coordination impairment as measured in rota-rod test.

It is worth noting that none of the compounds caused coordination impairments and myorelaxation as measured in the rota-rod and chimney tests (Figure 9 and Figure 10). Effects of the tested compounds on motor coordination evaluated in chimney (Figure 9) and rota-rod test (Figure 10). There were no significant changes in motor coordination of mice in both tests.

These results show the lack of neurotoxicity, and muscle relaxant potency of the new compounds, what is important, because coordination impairments can affect reliability of the other tests results [18].

Effects of compounds **3a**, **3e** and **3m** on the PTZ-induced clonic seizures, tonic convulsions and death of mice is shown in Table 2. Fisher exact tests showed that compound **3e** at the dose of 0.1 ED_50_ protected mice from clonic seizures.

## 3. Materials and Methods

### 3.1. General Information

All commercial reagents and solvents were purchased from Sigma-Aldrich Corp. (St.Louis, MO, USA) and used without purification. Reactions were routinely monitored by thin-layer chromatography (TLC) in silica gel (60 F_254_ plates Merck, Darmstadt, Germany) and the products were visualized with ultraviolet light of 254 nm wavelength. All NMR spectra were acquired on an AVANCE III 300 MHz spectrometer (Bruker Bioscience, Billerica, MA, USA) equipped with a BBO Z-gradient probe. Spectra were recorded at 25 °C using DMSO as a solvent with a non-spinning sample in 5 mm NMR-tubes. MS spectra were recorded on a Bruker microTOF-Q II (Bruker Bioscience, Billerica, MA, USA) and processed using Compass Data Analysis software (Brucker ASX, Karlsruhe, Germany). The elementary analysis was performed with the application of a Perkin-Elmer analyzer (Perkin Elmer Inc., Waltham, MA USA). Melting points were determined with a Boetius apparatus (Jena, Germany).

### 3.2. General Procedure for the Synthesis of Compounds ***3a**–**m***

The appropriate isocyanate **2** (0.01 mol) was dissolved in dichloromethane (25 mL) under an atmosphere of dry nitrogen and added to a solution of the free base of 1-alkyl-4-aryl-4,5-dihydro-1*H*-imidazo-2-amines **1a**–**c** (0.01 mol) dissolved in dichloromethane (50 mL). The reaction was continued for 8 h at room temperature. Solvent was removed by distillation and the rubber-like residue was treated with warm propan-2-ol. The solid 1-(1-alkyl-4-aryl-4,5-dihydro-1*H*-imidazo)-3-substituted urea derivatives **3a**–**m** were filtrated out and purified by crystallization from propan-2-ol.

#### 3.2.1. 1-(1-Methyl-4-phenyl-4,5-dihydro-1*H*-imidazo)-3-benzoilourea (**3a**)

From **1a** (1.75 g) and **2** (1.47 g) **3a** (1.19 g, 37% yield) was obtained as a white crystalline solid, mp 165–166 °C; ^1^H-NMR (DMSO-*d*_6_): δ(ppm) = 9.85 (s, 1H, NH); 8.30 (s, 1H, NH); 7.83–7.32 (m, 10H, H-Ar); 4.67 (t, 1H, CH, *J* = 8.2 Hz); 3.34–3.29 (m 2H, CH_2_); 2.66 (s, 3H, CH_3_). ^13^C-NMR (DMSO-*d*_6_): δ(ppm) = 161.5 (C=O), 160.9 (C=O), 149.2 (imidazolidine-C-2), 133.0, 131.5, 131.0, 129.2, 128.5, 128.3, 128.0, 123.1, 121.5 (C-Ar), 61.8 (imidazolidine-C-4), 50.2 (imidazolidine-C-5), 29.3 (CH_3_). EIMS *m*/*z* 323.3 [M + H]^+^. HREIMS (*m*/*z*): 322.0235 [M^+^] (calcd for C_18_H_18_N_4_O_2_ 322.3740); Anal. Calcd for: C_18_H_18_N_4_O_2_: C, 67.06; H, 5.62; N, 17.38. Found C, 67.25; H, 5.78; N, 17.17.

#### 3.2.2. 1-(1-Methyl-4-phenyl-4,5-dihydro-1*H*-imidazo)-3-(4-acetylophenyl)urea (**3b**)

From **1a** (1.75 g) and **2** (1.61 g) **3b** (2.35 g, 70% yield) was obtained as a white crystalline solid, mp 172–173 °C; ^1^H-NMR (DMSO-*d*_6_): δ(ppm) = 9.24 (s, 1H, NH); 8.52 (s, 1H, NH); 7.62–7.31 (m, 9H, H-Ar); 4.68–4.65 (m, 1H, CH); 3.91–3.80 (m, 2H, CH_2_); 2.49 (s, 3H, CH_3_); 2.30 (s, 3H, CH_3_). ^13^C-NMR (DMSO-*d*_6_): δ(ppm) = 165.3 (C=O), 162.5 (C=O), 149.1 (imidazolidine-C-2), 133.2, 130.9, 130.5, 129.1, 128.7, 127.9, 127.6, 122.2 (C-Ar), 62.1 (imidazolidine-C-4), 50.4 (imidazolidine-C-5), 26.8 (CH_3_), 21.2 (CH_3_),. EIMS *m*/*z* 337.5 [M + H]^+^. HREIMS (*m*/*z*): 336.1730 [M^+^] (Calcd for C_19_H_20_N_4_O_2_ 336.4010); Anal. Calcd for: C_19_H_20_N_4_O_2_: C, 67.84; H, 5.99; N, 16.66. Found C, 67.69; H, 5.87; N, 16.49.

#### 3.2.3. 1-(1-Methyl-4-phenyl-4,5-dihydro-1*H*-imidazo)-3-cyclopenthylurea (**3c**)

From **1a** (1.75 g) and **2** (1.11 g) **3c** (1.34 g, 47% yield) was obtained as a white crystalline solid, mp 169–170 °C; ^1^H-NMR (DMSO-*d*_6_): δ(ppm) = 8.16 (s, 1H, NH); 7.33–7.01 (m, 5H, H-Ar); 6.79 (s, 1H, NH); 4.38–4.31 (t, 1H, CH, *J* = 8.5 Hz); 3.89–3.80 (m, 2H, CH_2_); 3.16-3.05 (t, 1H, CH, *J* = 9 Hz); 2.19 (s, 3H, CH_3_); 1.77–1.61 (m, 4H, 2× CH_2_); 1.50–1.43 (m, 4H, 2x CH_2_). ^13^C-NMR (DMSO-*d*_6_): δ(ppm) = 162.2 (C=O), 149.1 (imidazolidine-C-2), 120.5, 119.0, 118.8, 116.8, 114.4 (C-Ar), 64.0 (imidazolidine-C-4), 53.5 (CH), 49.1 (imidazolidine-C-5), 38.0 (CH_2_), 36.3 (CH_2_), 31.5 (CH_2_), 29.0 (CH_3_), 22.9 (CH_2_). EIMS *m*/*z* 287.3 [M + H]^+^. HREIMS (*m*/*z*): 286.1911 [M^+^] (Calcd for C_16_H_22_N_4_O 286.3840); Anal. Calcd for: C_16_H_22_N_4_O: C, 67.10; H, 7.74; N, 19.56. Found C, 67.26; H, 7.71; N, 19.50.

#### 3.2.4. 1-(1-Methyl-4-phenyl-4,5-dihydro-1*H*-imidazo)-3-(2-chloroethyl)urea (**3d**)

From **1a** (1.75 g) and **2** (1.05 g) **3d** (0.95 g, 30% yield) obtained as a white crystalline solid, mp 206–208 °C; ^1^H-NMR (DMSO-*d*_6_): δ(ppm) = 9.96 (s, 1H, NH); 8.67 (s, 1H, NH); 7.55–7.01 (m, 5H, H-Ar); 4.81–4.73 (t, 1H, CH, *J* = 8.2 Hz); 4.51–4.30 (m. 1H, CH); 3.81–3.54 (m, 6H 3× CH_2_); 2.21 (s, 3H, CH_3_). ^13^C-NMR (DMSO-*d*_6_): δ(ppm) = 161.9 (C=O), 149.5 (imidazolidine-C-2), 227.7, 123.6, 119.5, 118.1, 118.0 (C-Ar), 53.5 (imidazolidine-C-4), 48.0 (imidazolidine-C-5), 41.2 (CH_2_), 34.1 (CH_2_), 25.3 (CH_3_). EIMS *m*/*z* 281.3 [M + H]^+^. HREIMS (*m*/*z*): 280.3110 [M^+^] (Calcd for C_13_H_17_N_4_ ClO 280.7680); Anal. Calcd for: C_13_H_17_N_4_ClO: C, 55.61; H, 6.10; N, 19.96; Cl, 12.63. Found C, 55.39; H, 6.18; N, 19.79; Cl, 12.51.

#### 3.2.5. 1-[1-Methyl-4-(4methylphenyl)-4,5-dihydro-1*H*-imidazo]-3-benzoilourea (**3e**)

From **1b** (1.89 g) and **2** (1.47 g) **3e** (2.55 g, 76% yield) was obtained as a white crystalline solid, mp 150–152 °C; ^1^H-NMR (DMSO-*d*_6_): δ(ppm) = 9.90 (s, 1H, NH); 8.32 (s, 1H, NH); 7.85–7.24 (m, 9H, H-Ar); 4.69–4.63 (m, 1H, CH); 3.97–3.91 (m, 2H, CH_2_); 2.65 (s, 3H, CH_3_); 2.33 (s, 3H, CH_3_). ^13^C-NMR (DMSO-*d*_6_): δ(ppm) = 165.0 (C=O), 162.1 (C=O), 149.1 (imidazolidine-C-2), 133.0, 131.5, 131.2, 130.8, 129.9, 129.5, 127.3, 127.1 (C-Ar), 61.7 (imidazolidine-C-4), 50.1 (imidazolidine-C-5), 29.3 (CH_3_), 21.2 (CH_3_). EIMS *m*/*z* 337.5 [M + H]^+^. HREIMS (*m*/*z*): 336.1350 [M^+^] (Calcd for C_19_H_20_N_4_O_2_ 336.4010); Anal. Calcd for: C_19_H_20_N_4_O_2_: C, 67.84; H, 5.99; N, 16.66. Found C, 67.71; H, 6.04; N, 16.79.

#### 3.2.6. 1-[1-Methyl-4-(4methylphenyl)-4,5-dihydro-1*H*-imidazo]-3-(4-acetylophenyl)urea (**3f**)

From **1b** (1.89 g) and **2** (1.61 g) **3f** (2.52 g, 72% yield) was obtaines as a white crystalline solid, mp 160–162 °C; ^1^H-NMR (DMSO-*d*_6_): δ(ppm) = 9.36 (s, 1H, NH); 8.28 (s, 1H, NH); 7.87–7.24 (m, 8H, H-Ar); 4.66–4.60 (m, 1H, CH); 3.95–3.89 (m, 2H, CH_2_); 2.68 (s, 3H, CH_3_); 2.51 (s, 3H, CH_3_); 2.33(s, 3H, CH_3_). ^13^C-NMR (DMSO-*d*_6_) = δ(ppm): 163.8 (C=O), 162.9 (C=O), 149.6 (imidazolidine-C-2), 131.2, 130.0, 129.1, 127.5, 121.9, 120.8, 119.5, 119.1, 117.9, 117.4 (C-Ar), 61.8 (imidazolidine-C-4), 50.1 (imidazolidine-C-5), 26.9 (CH_3_), 25.3 (CH_3_), 21.31 (CH_3_). EIMS *m*/*z* 351.1 [M + H]^+^. HREIMS (*m*/*z*): 350.17230 [M^+^] (Calcd for C_20_H_22_N_4_O_2_ 350.4280); Anal. Calcd for: C_20_H_22_N_4_O_2_: C, 68.55; H, 6.33; N, 15.99. Found C, 68.26; H, 6.39; N, 15.84.

#### 3.2.7. 1-[1-Methyl-4(4-methylphenyl)-4,5-dihydro-1*H*-imidazo]-3-cyclopenthylurea (**3g**)

From **1b** (1.89 g) and **2** (1.11 g) **3g** (1.74g, 58% yield) was obtained as a white crystalline solid, mp 173–174 °C; ^1^H-NMR (DMSO-*d*_6_): δ(ppm) = 8.15 (s, 1H, NH); 7.23–7.19 (m, 4H, H-Ar); 6.41 (s, 1H, NH); 4.48–4.46 (t, 1H, CH, *J* = 8.5 Hz); 3.81–3.89 (m, 2H, CH_2_); 3.18–3.15 (t, 1H, CH, *J* = 9.0 Hz); 2.55 (s, 3H, CH_3_); 2.31 (s, 3H, CH_3_); 1.75–1.69 (m, 4H, 2× CH_2_); 1.47–1.40 (m, 4H, 2× CH_2_). ^13^C-NMR (DMSO-*d*_6_): δ(ppm) = 162.2 (C=O), 148.1 (imidazolidine-C-2), 130.1, 129.9, 128.4, 128.1, 127.4 (C-Ar), 61.9 (imidazolidine-C-4), 49.9 (imidazolidine-C-5), 51.5 (CH), 38.2 (CH_2_), 36.8 (CH_2_), 32.9 (CH_2_), 29.9 (CH_3_), 23.8 (CH_2_), 21.2 (CH_3_). EIMS *m*/*z* 301.3 [M + H]^+^. HREIMS (*m*/*z*): 300.1170 [M^+^] (Calcd for C_17_H_24_N_4_O 300.4110); Anal. Calcd for: C_17_H_24_N_4_O: C, 67.97; H, 8.05; N, 18.65. Found C, 67.79; H, 8.18; N, 18.59.

#### 3.2.8. 1-[1-Methyl-4(4-methylphenyl)-4,5-dihydro-1*H*-imidazo]-3-(2-chloroethyl)urea (**3h**)

From **1b** (1.89 g) and **2** (1.05 g) **3h** (1.06 g, 32% yield) was obtained as a white crystalline solid, mp 178–180 °C; ^1^H-NMR (DMSO-*d*_6_): δ(ppm) = 9.56 (s, 1H, NH); 8.71 (s, 1H, NH); 7.44–6.91 (m, 4H, H-Ar); 4.79–4.60 (t, 1H, CH, *J* = 8.1 Hz); 4.21–4.17 (m. 1H, CH); 3.96–3.87 (m, 6H 3× CH_2_); 2.39 (s, 3H, CH_3_); 2.11 (s, 3H, CH_3_). ^13^C-NMR (DMSO-*d*_6_): δ(ppm) = 163.9 (C=O), 149.0 (imidazolidine-C-2), 127.9, 120.7, 120.6, 119.5, 119.1, 114.9 (C-Ar), 52.5 (imidazolidine-C-4), 48.7 (imidazolidine-C-5), 42.2 (CH_2_), 36.1 (CH_2_), 23.8 (CH_3_), 20.9 (CH_3_). EIMS *m*/*z* 295.5 [M + H]^+^. HREIMS (*m*/*z*): 294.2610 [M^+^] (Calcd for C_14_H_19_N_4_ClO 294.7950); Anal. Calcd for: C_14_H_19_N_4_ClO: C, 57.04; H, 6.50; N, 19.10; Cl, 12.03. Found C, 57.11; H, 6.42; N, 19.04; Cl, 12.09.

#### 3.2.9. 1-(1-Ethyl-4-phenyl-4,5-dihydro-1*H*-imidazo)-3-benzoilourea (**3i**)

From **1c** (1.89 g) **2** (1.47 g) **3i** (1.74 g, 52% yield) was obtained as a white crystalline solid, mp 136–138 °C; ^1^H-NMR (DMSO-*d*_6_): δ(ppm) = 9.83 (s, 1H,NH); 8.62 (s, 1H, NH); 7.80–7.44 (m, 10H, H-Ar); 4.71–4.60 (m, 1H, CH); 3.87–3.81 (m, 2H, CH_2_); 2.70–2.65 (q, 2H, CH_2_, *J* = 6.9 Hz); 2.18–2.05 (t, 3H, CH_3_, *J* = 7.1 Hz). ^13^C-NMR (DMSO-*d*_6_): δ(ppm) = 164.0 (C=O), 160.5 (C=O), 149.7 (imidazolidine-C-2), 133.5, 131.5, 131.0, 129.2, 127.5, 127.3, 126.0, 122.1, 120.5 (C-Ar), 60.8 (imidazolidine-C-4), 51.2 (imidazolidine-C-5), 34.2 (CH_2_), 23.6 (CH_3_),. EIMS *m*/*z* 337.5 [M + H]^+^. HREIMS (*m*/*z*): 336.3510 [M^+^] (Calcd for C_19_H_20_N_4_O_2_ 336.4010); Anal. Calcd for: C_19_H_20_N_4_O_2_: C, 67.84; H, 5.99; N, 16.66. Found C, 67.91; H, 5.86; N, 16.79.

#### 3.2.10. 1-(1-Ethyl-4-phenyl-4,5-dihydro-1*H*-imidazo)-3-(4-acetylophenyl)urea (**3j**)

From **1c** (1.89 g) and **2** (1.61 g) **3j** (1.99 g, 57% yield) was obtained as a white crystalline solid, mp 153–154 °C; ^1^H-NMR (DMSO-*d*_6_): δ(ppm) = 9.34 (s, 1H, NH); 8.27 (s, 1H, NH); 7.83–7.35 (m, 9H, H-Ar); 4.89–4.84 (m, 1H, CH); 3.31–3.29 (m, 2H, CH_2_); 2.76–2.71 (q, 2H, CH_2,_ *J* = 7.0 Hz); 2.49 (s, 3H, CH_3_); 0.98–0.95 (t, 3H, CH_3_, *J* = 7.1 Hz). ^13^C-NMR (DMSO-*d*_6_)= δ(ppm) = 162.8 (C=O), 162.1 (C=O), 146.6 (imidazolidine-C-2), 130.1, 129.9, 129.8, 129.6, 129.4, 120.1, 128.7, 1`27.7, 127.3, 118.8, 117.4 (C-Ar), 58.9 (imidazolidine-C-4), 41.1 (imidazolidine-C-5), 36.6 (CH_2_), 12.5 (CH_3_), 11.9 (CH_3_). EIMS *m*/*z* 351.1 [M + H]^+^. HREIMS (*m*/*z*): 350.1150 [M^+^] (Calcd for C_20_H_22_N_4_O_2_ 350.4280); Anal. Calcd for: C_20_H_22_N_4_O_2_: C, 68.55; H, 6.33; N, 15.99. Found C, 68.65; H, 6.26; N, 15.81.

#### 3.2.11. 1-(1-Ethyl-4phenyl-4,5-dihydro-1*H*-imidazo)-3-cyclopenthylurea (**3k**)

From **1c** (1.89 g) **2** (1.11 g) **3k** (1.71 g, 57% yield) was obtained as a white crystalline solid, mp 176–177 °C; ^1^H-NMR (DMSO-*d*_6_): δ(ppm) = 9.15 (s, 1H, NH); 7.40–7.29 (m, 5H, H-Ar); 6.89 (s, 1H, NH); 4.51–4.48 (t, 1H, CH, *J* = 8.4 Hz); 3.65–3.40 (m, 4H, CH_2_); 3.20–3.11 (t, 1H, CH, *J* = 9.0 Hz); 2.19–2.11 (t, 3H, CH_3_, *J* = 7.1 Hz); 1.75–1.61 (m, 4H, 2× CH_2_); 1.47–1.40 (m, 4H, 2× CH_2_). ^13^C-NMR (DMSO-*d*_6_): δ(ppm) = 162.0 (C=O), 148.8 (imidazolidine-C-2), 123.1, 120.9, 118.8, 118.5, 117.0 (C-Ar), 61.9 (imidazolidine-C-4), 51.5 (CH), 49.9 (imidazolidine-C-5), 39.0 (CH_2_), 38.2 (CH_2_), 36.8 (CH_2_), 32.9 (CH_2_), 29.9 (CH_3_), 26.0 (CH_2_), 24.6 (CH_3_). EIMS *m*/*z* 301.3 [M + H]^+^. HREIMS (*m*/*z*): 300.3920 [M^+^] (Calcd for C_17_H_24_N_4_O 300.4110); Anal. Calcd for: C_17_H_24_N_4_O: C, 67.97; H, 8.05; N, 18.65. Found C, 67.90; H, 8.16; N, 18.60.

#### 3.2.12. 1-(1-Ethyl-4-phenyl-4,5-dihydro-1*H*-imidazo)-3-(2-chloroethyl)urea (**3l**)

From **1c** (1.89 g) and **2** (1.05 g) **3l** (2.31 g, 70% yield)was obtained as a white crystalline solid, mp 203–205 °C; ^1^H-NMR (DMSO-*d*_6_): δ(ppm) = 10.06 (s, 1H, NH); 8.49 (s, 1H, NH); 7.48–7.41 (m, 5H, H-Ar); 4.91–4.88 (t, 1H, CH, *J* = 8.4 Hz); 4.41–4.37 (m, 1H, CH); 3.85–3.71 (m, 6H 3× CH_2_); 3.51–3.38 (t, 3H, CH_3_, *J* = 7.0 Hz). ^13^C-NMR (DMSO-*d*_6_): δ(ppm) = 162.9 (C=O), 148.5 (imidazolidine-C-2), 127.7, 129.6, 129.5, 128.1, 128.0 (C-Ar), 51.5 (imidazolidine-C-4), 44.9 (imidazolidine-C-5), 43.1 (CH_2_), 42.2 (CH_2_), 39.2 (CH_2_), 30.6 (CH_3_). EIMS *m*/*z* 295.3 [M + H]^+^. HREIMS (*m*/*z*): 294.1170 [M^+^] (Calcd for C_14_H_19_N_4_ClO 294.795); Anal. Calcd for: C_14_H_19_N_4_ClO: C, 57.04; H, 6.50; N, 19.10; Cl, 12.03. Found C, 57.15; H, 6.55; N, 19.06; Cl, 12.19.

#### 3.2.13. 1-(1-Ethyl-4-phenyl-4,5-dihydro-1*H*-imidazo)-3-(4-etoxycarbonylphenyl)urea (**3m**)

From **1c** (1.89 g) and **2** (1.91 g) **3m** (2.39 g, 63% yield) was obtained as a white crystalline solid, mp 160–161 °C; ^1^H-NMR (DMSO-*d*_6_): δ(ppm) = 9.30 (s, 1H, NH); 8.27 (s, 1H, NH); 7.93–7.38 (m, 9H, H-Ar); 4.84–4.81 (m, 1H, CH); 3.67–3.33 (m, 2H, CH_2_); 1.46–1.30 (m, 4H, 2× CH_2_); 1.29–1.20 (t, 3H, CH_3_, *J* = 7.0 Hz); 0.97–0.95 (t, 3H, CH_3_, *J* = 7.2 Hz); ^13^C NMR (DMSO-*d*_6_) = δ(ppm): 166.1 (C=O), 162.3 (C=O), 156.4 (imidazolidine-C-2), 130.4, 129.4, 129.1, 128.7, 127.2, 124.8, 123.4, 122.1, 119.1, 117.5 (C-Ar), 11.8 (CH_3_), 58.9 (imidazolidine-C-4), 41.0 (imidazolidine-C-5), 38.1 (CH_2_), 36.5 (CH_2_), 12.6 (CH_3_). EIMS *m*/*z* 381.5 [M + H]^+^. HREIMS (*m*/*z*): 380.3690 [M^+^] (Calcd for C_21_H_24_N_4_O_3_ 380.4550); Anal. Calcd for: C_21_H_24_N_4_O_3_: C, 66.29; H, 6.36; N, 14.73. Found C, 66.16; H, 6.21; N, 14.59.

### 3.3. Molecular Modeling

The investigated compounds were modeled using the LigPrep protocol from the Schrödinger Suite [43]. In order to sample different protonation states of ligands in physiological pH, Epik module was used [44]. The compounds were further optimized using Hartree-Fock approach and 6–31 g(d, p) basis set of Spartan 10 [45]. Parameters to evaluate drug-likeness were calculated using VegaZZ v. 3.0.1 [46] (number of atoms), Discovery Studio v. 3.1. [47] (molar mass, number of rings, lipophilicity, number of rotatable bonds), ACDLabs (molar refractivity, number of hydrogen bond donors and acceptors), and the Schrödinger Suite (a number of rigid bonds) as described previously [2,6,7,8]. ADMET parameters were calculated with Discovery Studio 3.1 (solubility, blood-brain permeation) or Osiris Property Explorer [48] (toxicity risks) as previously described [2,6,7,8].

### 3.4. Pharmacology

The experiments were performed on male Albino Swiss mice (18–30 g). 8–10 animals were maintained in a cage, at room temperature of 22 ± 1 °C, on a natural dark-light cycle of 12 h each, and had *ad libitum* access to pelleted rodent chow (LSM, Motycz, Poland) and tap water. The mice were acclimated for 1 week prior to use. All behavioural experiments were carried out, according to the National Institute of Health Guidelines for the Care and Use of Laboratory Animals and to the European Community Directive for the Care and Use of Laboratory of 24 November 1986 (86/609/EEC), and approved by the Local Ethics Committee for Animal Experimentation (14/2014).

In behavioural experiments urea derivatives **3a**, **3e** and **3m** were administered intraperitoneally (i.p.), suspended in aqueous solution of 0.5% methylcellulose (tylose) and were injected 60 min before tests. In “writhing” procedure, the tested substances were injected subcutaneously (s.c.) and the acetic acid (0.6%) was administered i.p. All substances were given in a volume of 0.1 mL per 10 g body mass. The control animals received an equivalent volume of the solvent at the respective time before the test.

All tests performed, suggested by Vogel and Vogel [49], are generally accepted for the investigation of the central activity by behavioral methods. The acute toxicity of the compound was assessed according to Litchfield and Wilcoxon method [13], as the ED_50_ calculated as “the loss of righting reflex” within 48 h. After i.p. administration of compounds tested, observation of mice was carried out in the following time intervals: 15, 30, 60 and 180 min, 24 and 48 h. in pharmacological experiments, each compound was injected in doses equivalent to 0.1, 0.05 and 0.025 ED_50_ (Table 1).

In addition, the activity of compounds was assessed in the tests described below:

Locomotor activity was measured in a photocell apparatus. The number of photocell interruptions of each mouse for a total period of 30 min was recorded both as spontaneous activity and amphetamine-induced hyperactivity (mice received amphetamine 30 min before the test, 5 mg/kg, s.c.).

Nociceptive reactions were studied in the acetic acid “writhing” test [50]. In this test the number of writhing episodes was measured for 10 min, starting 5 min after i.p. administration of 0.6% acetic acid solution.

In this test, the influence of naloxone on the antinociceptive effect of the compounds was also assessed. For this purpose, naloxone (Nx, 5 mg/kg, s.c.) was applied 30 min before acetic acid solution injection. The dose of Nx was chosen based on the previous results [50].

Motor coordination was evaluated in the rota-rod [51] and chimney tests [52]. In the first test, the motor impairments, defined as the inability to remain on the rotating rod (a constant speed of 18 rpm) for 1 min were measured, and the mean time spent on the rota-rod was counted for each mouse. In the second test, the motor impairments were assessed as the inability of mice to climb backwards up the tube (3 cm in inner diameter, 25 cm long) within 60 s. Before the tests, the animals were trained once a day for 3 days. The animals able to stay on the rotating rod or to leave the chimney for 60 s were approved for experiments.

Body temperature in normothermic mice was measured in the rectum of animals with a thermistor thermometer during the total period 180 min (60 min before and 120 min after the tested compound injection). The average of the first two measurements (60 and 30 min before drug administration) was determined as an initial temperature (*t*_i_). The final temperature (*t*_f_) was measured 30, 60, 90 and 120 min after the injection of the tested compounds at a dose of 0.1 ED50. The changes in body temperature (Δt) were calculated according to the formula: Δt = *t*_f_ − *t*_i_.

Anticonvulsive activity: An injection of pentylenetetrazole (PTZ 110 mg/kg, s.c.) was given to elicit seizure-like activity in mice. Convulsions in various treatment groups were evaluated for a period of 60 min, immediately after PTZ administration, in the glass chambers as the number of mice with clonic seizures, tonic convulsions and dead animals. 

’Head-twitch’ responses (HTR) after 5-hydroxy-l-tryptophan (l-5-HTP) administration were estimated according to Corne *et al*. [26]. Mice received l-5-HTP (230 mg/kg, i.p.) and the number of HTR was recorded in 6 two-minute intervals (4–6, 14–16, 24–26, 34–36, 44–46, 54–56 min) during 1 h.

To avoid subjective estimation two experienced observers were engaged in all conducted experiments. Experimenters were not aware what observed group of mice was injected.

The results were calculated by Fisher exact tests (PTZ-induced seizures), two-way analysis of variance (ANOVA) (body temperature test) and one-way ANOVA (other tests) followed by Dunnett’s multiple comparison *post hoc* tests. Specific paired comparison was performed with a Student’s *t*-test when necessary. All data are expressed as mean ± standard errors (SEM). In all comparisons, *p <* 0.05 was used as the criterion for statistical significance.

## 4. Conclusions

In this study we obtained 13 novel 1-(1,4-alkylaryldisubstituted-4,5-dihydro-1*H*-imidazo)-3-substituted urea derivatives and evaluated for their CNS activity. The mechanism of action of compound **3m** seems to be connected with serotoninergic transmission due to the decrease in body temperature and the reduction of HTR. Moreover, **3e** showed antinociceptive (not mediated by opioid system) and anticonvulsant activity (protection against seizures induced by PTZ). Both compounds, **3e** and **3m**, appear to have interactions with catecholamine system (inhibition of spontaneous and amphetamine-induced activity). It should be emphasized that none of tested molecules impaired motor coordination and caused myorelaxation, which may suggest low neurotoxicity.

In conclusion, the new urea derivatives exerted significant activities in the performed pharmacological tests, although presented results show preliminary estimation, thus, need to be extended for identification and understanding the complete pharmacological profile of the examined compounds.

## Figures and Tables

**Table 1:** ED_50_ values and doses of urea derivatives used in behavioural experiments.

Compound	ED_50_ (mg/kg)	Dose
3a	330.2	0.1 ED_50_
3e	1133.7	0.1; 0.05; 0.025 ED_50_
3m	2000	0.1; 0.05; 0.025 ED_50_

**Table 2:** The effect of compounds **3a**, **3e** and **3m** on the PTZ-induced clonic seizures, tonic convulsions and death of mice.

Compound	Clonic Seizures ^#^	Tonic Convulsions ^#^	Mortality ^^^
0.1 ED_50_ of **3a**	8/10	4/10	4/10
0.1 ED_50_ of **3e**	6/10 *	5/10	3/10
0.05 ED_50_ of **3e**	9/10	5/10	5/10
0.1 ED_50_ of **3m**	9/10	8/10	7/10
Control (PTZ 110 mg/kg)	12/12	8/12	7/12

^#^ Number of mice reacting/animals tested; ^^^ number of dead mice/animals tested, * *p <* 0.05 *vs.* control (Fisher exact test).

## Supplementary Materials

The following are available online at http://www.mdpi.com/1420-3049/21/5/582/s1, Table S1. Parameters for drug-likeness estimation; Table S2. ADMET parameters of the studied compounds; Figure S1. Evaluation of ADMET properties of the studied compounds.
