# Supplementary material for: Synthesis and Pharmacological Evaluation of Novel 1-(1,4-Alkylaryldisubstituted-4,5-dihydro-1H-imidazo)-3-substituted Urea Derivatives
Source: Molecules. 2016 Apr 30;21(5):582. doi: 10.3390/molecules21050582 (PMC6274338; doi:10.3390/molecules21050582)
Supplement: Supplementary file 1 [file molecules-21-00582-s001.pdf]

# Supplementary Materials: Synthesis and pharmacological evaluation of novel 1-(1,4-alkylaryldisubstituted-4,5-dihydro-1*H*-imidazo)-3-substituted urea derivatives

**Elżbieta Szacoń, Marzena Rządkowska, Agnieszka A. Kaczor, Ewa Kędzierska, Jolanta Orzelska-Górka, Sylwia Fidecka and Dariusz Matosiuk**

## Estimation of Drug-likeness

The descriptors applied for estimation of drug-likeness are presented in Table S1. Drug-likeness was assessed using Lipinski's rule as well as the placement of the investigated compounds in the chemical space determined by the databases of the pharmacologically active compounds (CMC, Comprehensive Medicinal Chemistry Database, containing about 7,000 compounds and MDDR, MACCS-II Drug Data Report, containing about 100,000 compounds) according to the methodology of PREADMET [1] service as described previously [2,3–9]. Regarding Lipinski's rule, all the compounds possess the molar mass below 500, the number of hydrogen bond donors below 5, the number of hydrogen bond acceptors below 10, and the lipophilicity below 5.

Regarding subsequent criteria of drug-likeness, most compounds collected in the CMC database has lipophilicity from -0.4 to 5.6, molar refractivity in the range of 40–130 the number of atoms from 20 to 70 and molar mass from 160 to 480 [2,3–9]. All the investigated compounds fulfill all these criteria. Concerning the compounds in MDDR database, the drug-like substances have the number of rings equal or greater than 3, the number of rigid bonds equal or greater than 18, and the number of rotatable bonds equal or greater than 6 [2,3–9]. Compounds **3a**-**3m** have too few rings and too few rotatable bonds. The criterion concerning rigid bonds is fulfilled by all the compounds.

**Table S1.** Parameters for drug-likeness estimation. HBD - a number of hydrogen bond donors; HBA -a number of hydrogen bond acceptors.

| Comp. | Molar Mass | logP | HBD | HBA | Atoms | Molar Refractivity, $cm^3$ | Rings | Rigid Bonds | Rotatable Bonds |
|---|---|---|---|---|---|---|---|---|---|
| **3a** | 322.36 | 2.49 | 2 | 6 | 42 | 92.49 | 2 | 41 | 3 |
| **3b** | 336.38 | 2.36 | 2 | 6 | 45 | 96.91 | 2 | 43 | 4 |
| **3c** | 286.37 | 2.45 | 2 | 5 | 43 | 82.42 | 2 | 42 | 3 |
| **3d** | 280.75 | 1.71 | 2 | 5 | 36 | 75.74 | 2 | 33 | 4 |
| **3e** | 336.38 | 2.98 | 2 | 6 | 45 | 96.91 | 2 | 44 | 3 |
| **3f** | 350.41 | 2.85 | 2 | 6 | 48 | 101.33 | 2 | 46 | 4 |
| **3g** | 300.39 | 2.93 | 2 | 5 | 46 | 86.84 | 2 | 45 | 3 |
| **3h** | 294.78 | 2.20 | 2 | 5 | 39 | 80.16 | 2 | 36 | 4 |
| **3i** | 336.38 | 2.84 | 2 | 6 | 45 | 97.1 | 2 | 43 | 4 |
| **3j** | 378.42 | 2.58 | 2 | 7 | 50 | 106.98 | 2 | 50 | 5 |
| **3k** | 300.39 | 2.80 | 2 | 5 | 46 | 87.03 | 2 | 44 | 4 |
| **3l** | 294.78 | 2.06 | 2 | 5 | 39 | 80.35 | 2 | 35 | 5 |
| **3m** | 380.44 | 3.18 | 2 | 7 | 52 | 107.52 | 2 | 37 | 7 |

## Prediction of ADMET Properties

In order to facilitate the selection of compounds for pharmacological activity assessment, some ADMET parameters were calculated (Table S2). The plot presented in Figure S1 confirms that all of the tested compounds possess reasonably favorable ADMET properties. All compounds are well

absorbed (Figure. S1) and well soluble in water as they have of logS over -4 (with the exception of compounds **3e** and **3m**) [10]. Compounds **3b**, **3d**, **3f**, **3h**, **3j**, **3l** were predicted to have low mutagenicity and/or tumorigenicity risk (Table S2). Compounds **3d**, **3h** **3l** may have some reproductive effects (Table S2). No possible irritating effects were predicted.

**Table S2.** ADMET parameters of the studied compounds. S - solubility.

| Comp. | logS | Mutagenicity | Tumorigenicity | Irritating Effects | Reproductive Effects |
|---|---|---|---|---|---|
| **3a** | -3.75 | 1.0 | 1.0 | 1.0 | 1.0 |
| **3b** | -3.48 | 0.6 | 0.8 | 1.0 | 1.0 |
| **3c** | -3.38 | 1.0 | 1.0 | 1.0 | 1.0 |
| **3d** | -2.58 | 0.6 | 0.6 | 1.0 | 0.6 |
| **3e** | -4.21 | 1.0 | 1.0 | 1.0 | 1.0 |
| **3f** | -3.94 | 0.6 | 0.8 | 1.0 | 1.0 |
| **3g** | -3.87 | 1.0 | 1.0 | 1.0 | 1.0 |
| **3h** | -3.07 | 0.6 | 0.6 | 1.0 | 0.6 |
| **3i** | -3.97 | 1.0 | 1.0 | 1.0 | 1.0 |
| **3j** | -3.84 | 1.0 | 0.6 | 1.0 | 1.0 |
| **3k** | -3.63 | 1.0 | 1.0 | 1.0 | 1.0 |
| **3l** | -2.83 | 0.6 | 0.6 | 1.0 | 0.6 |
| **3m** | -4.02 | 1.0 | 1.0 | 1.0 | 1.0 |

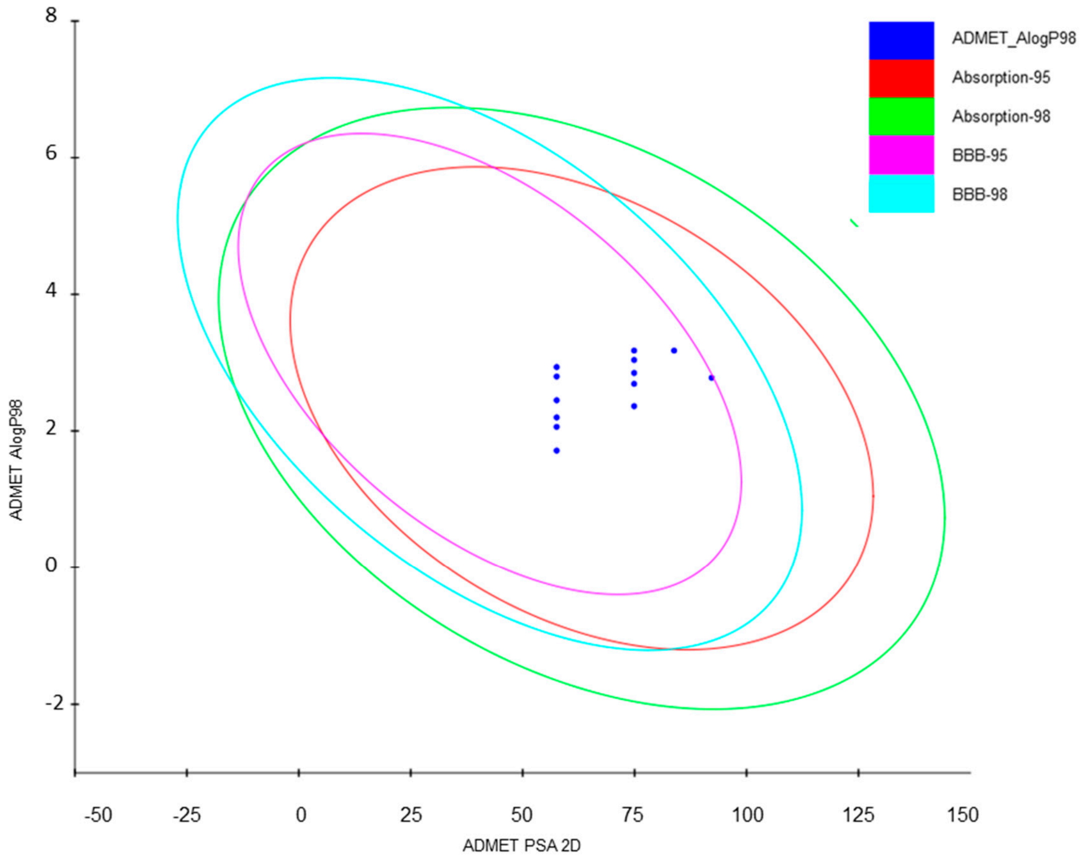


**Figure S1.** Evaluation of ADMET properties of the studied compounds.

## References

1. Lee, S.K.; Chang, G.S.; Lee, I.H.; Chung, J.E.; Sung, K.Y.; No. K.T. The PreADME:pc-based program for batch prediction of adme properties, In Proceedings of EuroQSAR, Istanbul, Turkey, 5–10 September 2004.

2. Rządkowska, M.; Szacoń, E.; Kaczor, A.A.; Fidecka, S.; Kędzierska, E.; Matosiuk, D. Synthesis, pharmacological activity and molecular modeling of 1-aryl-7-hydroxy-2,3-dihydroimidazo[1,2-a]pyrimidine-5(1*H*)-ones and their 6-substituted derivatives. *Med. Chem.* **2014**, *10*, 460–475.
3. Rządkowska, M.; Szacoń, E.; Kaczor, A.A.; Fidecka, S.; Kędzierska, E.; Matosiuk, D. Synthesis, central nervous system activity, and structure–activity relationship of 1-aryl-6-benzyl-7-hydroxy-2,3-dihydroimidazo[1,2-a]pyrimidine-5(1H)-ones. *Med. Chem. Res.* **2014**, *23*, 4221–4237.
4. Szacoń, E.; Rządkowska, M.; Kaczor, A.A.; Kędzierska, E.; Mazur, A.; Fidecka, S.; Matosiuk, D. Synthesis, central nervous system activity and structure-activity relationship of N-substituted derivatives of 1-arylimidazolidyn-2-ylideneurea and products of their cyclization. *J. Enzyme Inhib. Med. Chem*. **2015**, *30*, 746–760.
5. Szacoń, E.; Rządkowska, M.; Kaczor, A.A.; Kędzierska, E.; Mazur, A.; Fidecka, S.; Matosiuk D. Synthesis, central nervous system activity and structure-activity relationships of novel 1-(1-Alkyl-4-aryl-4,5-dihydro-1*H*-imidazo)-3-substituted urea derivatives. *Molecules* **2015**, *20*, 3821–3840.
6. Matosiuk, D.; Fidecka, S.; Antkiewicz-Michaluk, L.; Dybała, I.; Kozioł A.E. Synthesis and pharmacological activity of new carbonyl derivatives of 1-aryl-2-iminoimidazolidine. Part 1. Synthesis and pharmacological activity of chain derivatives of 1-aryl-2-iminoimidazolidine containing urea moiety. *Eur. J. Med. Chem*. **2001**, *36*, 783–797.
7. Matosiuk, D.; Fidecka, S.; Antkiewicz-Michaluk, L.; Dybała, I.; Kozioł A.E. Synthesis and pharmacological activity of new carbonyl derivatives of 1-aryl-2-iminoimidazolidine. Part 3. Synthesis and pharmacological activity of 1-aryl-5,6(1*H*)dioxo-2,3-dihydroimidazo[1,2-a]imidazoles. *Eur. J. Med. Chem.* **2002**, *37*, 845–853.
8. Matosiuk, D.; Fidecka, S.; Antkiewicz-Michaluk, L.; Lipkowski, J.; Dybała, I.; Kozioł, A.E. Synthesis and pharmacological activity of new carbonyl derivatives of 1-aryl-2-iminoimidazolidine: Part 2. Synthesis and pharmacological activity of 1,6-diaryl-5,7(1*H*)dioxo-2,3-dihydroimidazo[1,2-a][1,3,5]triazines. *Eur. J. Med. Chem*. **2002**, *37*, 761–772.
9. Sztanke, K.; Fidecka, S.; Kędzierska, E.; Karczmarzyk, Z.; Pihlaja, K.; Matosiuk, D. Antinociceptive activity of new imidazolidine carbonyl derivatives. Part 4. Synthesis and pharmacological activity of 8-aryl-3,4-dioxo-2*H*,8*H*-6,7-dihydroimidazo[2,1-c] [1,2,4]triazines. *Eur. J. Med. Chem*. **2005**, *40*, 127–134.
10. Osiris Property Explorer. Available from: http://www.organic-chemistry.org/prog/peo/logS.html (accessed on 30 April 2016).
